# Spatiotemporal Patterns of Five Small Heat Shock Protein Genes in *Hyphantria cunea* in Response to Thermal Stress

**DOI:** 10.3390/ijms242015176

**Published:** 2023-10-14

**Authors:** Shiyue Zhao, Yukun Liu, Hui Li, Zichun Li, Dejun Hao

**Affiliations:** 1 Co-Innovation Center for Sustainable Forestry in Southern China, Nanjing Forestry University, Nanjing 210037, China; zhaoshiyue0211@163.com (S.Z.); liuyukun@njfu.edu.cn (Y.L.); 15720616317@163.com (H.L.); leezichun@njfu.edu.cn (Z.L.); 2 College of Forestry, Nanjing Forestry University, Nanjing 210037, China

**Keywords:** *Hyphantria cunea*, small heat shock protein, expression profiles, high temperature, different developmental stages

## Abstract

*Hyphantria cunea* (Drury), a destructive polyphagous pest, has been spreading southward after invading northern China, which indicates that this insect species is facing a huge thermal challenge. Small heat shock proteins (sHSPs) function as ATP-independent molecular chaperones that protect insects from heat stress damage. In order to explore the role of sHSPs in the thermotolerance of *H. cunea*, five novel sHSP genes of *H. cunea* were cloned, including an orthologous gene (*HcHSP21.4*) and four species-specific sHSP genes (*HcHSP18.9*, *HcHSP20.1*, *HcHSP21.5*, and *HcHSP29.8*). Bioinformatics analysis showed that the proteins encoded by these five *HcHSPs* contained typical α-crystallin domains. Quantitative real-time PCR analysis revealed the ubiquitous expression of all *HcHSPs* across all developmental stages of *H. cunea*, with the highest expression levels in pupae and adults. Four species-specific *HcHSPs* were sensitive to high temperatures. The expression levels of *HcHSPs* were significantly up-regulated under heat stress and increased with increasing temperature. The expression levels of *HcHSPs* in eggs exhibited an initial up-regulation in response to a temperature of 40 °C. In other developmental stages, the transcription of *HcHSPs* was immediately up-regulated at 30 °C or 35 °C. *HcHSPs* transcripts were abundant in the cuticle before and after heat shock. The expression of *HcHSP21.4* showed weak responses to heat stress and constitutive expression in the tissues tested. These results suggest that most of the *HcHSPs* are involved in high-temperature response and may also have functions in the normal development and reproduction of *H. cunea*.

## 1. Introduction

Heat shock proteins (HSPs) are widely distributed in both eukaryotes and prokaryotes [1]. HSPs were first identified in the insect *Drosophila melanogaster* by Ritossa et al. [2]. They occur when organisms are subjected to stress, including starvation, extreme temperature, virus invasion, heavy metals, chemical agents, and UV light [3,4,5,6]. HSPs act as molecular chaperones that can protect proteins from irreversible denaturation and help proteins to refold correctly [7,8]. HSPs have been classified into five families based on molecular mass and homology: HSP100, HSP90, HSP70, HSP60, and small heat shock proteins (called sHSPs) [7,8].

Compared to the other HSP families, individual sHSPs show less homology [9], with greater diversity in molecular weight, protein structure, and biological function [10]. The sHSPs range in molecular weight from 12 to 43 kDa and consist of an α-crystal domain (ACD), a variable N-terminal, and a conserved C-terminal. The conserved ACD generally contains 80–100 amino acid residues, consisting of seven or eight antiparallel β-strands that form a β-sandwich structure. The N-terminal contains a specific structure consisting of approximately 55 amino acids, and the C-terminal of the sequence possesses a conserved I/V/L-X-I/V/L motif [11,12,13]. The sHSPs normally function as the first line of cellular defense in an ATP-independent manner, binding equal-molecular-weight substrate proteins such as oligomers [8,13,14]. Thus, the accumulation of denatured proteins is prevented from causing irreversible damage to cells [9,15].

sHSPs are common in insects and are involved in growth, development, and resistance to environmental stressors [8,16,17,18]. High temperature is an abiotic factor that can induce sHSP expression in insects [19]. When insects are exposed to extremely high temperatures, sHSPs protect proteins from misfolding, denaturation, and aggregation to maintain cellular proteostasis [8,20]. The expression of sHSPs can enhance heat adaptation, heat tolerance, and heat protection [5,8,21,22]. For example, the *HSP21* of *Antheraea pernyi* [23], *HSP21.8* of *Glyphodes pyloalis* [24], and *HSP22.2* of *Tribolium castaneum* [25] were all rapidly up-regulated after heat treatment in different trials. Also, the expression patterns of sHSPs in many insects can vary in different developmental stages or tissues. For example, *HSP21.3* and *HSP22.0* in *Choristoneura fumiferana* are most highly expressed in the head and cuticle, while most sHSPs are significantly up-regulated in the fat body after heat stress [26]. In *Bombyx mori*, *HSP19.1* and *HSP22.6* were highly expressed in the cuticle, head, and midgut, while *HSP20.1*, *HSP20.4*, and *HSP27.4* were highly expressed in the gonads [27]. These findings demonstrated that multiple sHSPs have different expression patterns and variable functions, which are related to the thermotolerance of insects.

The fall webworm, *Hyphantria cunea* Drury (Lepidoptera: Erebidae), is a quarantine pest that feeds on more than 600 plant species, including forest and fruit trees, shrubs, herbaceous plants, and crops [28,29,30]. *H. cunea* is native to North America. It spread to Eurasia in the 1940s and is now present in 32 countries worldwide [31]. In 1979, it invaded China via Dandong City, Liaoning Province, and caused significant economic and ecological damage [32]. Due to its polyphagy, adaptability, and high fecundity, *H. cunea* has spread rapidly in China. Invasion has proceeded from north to south, and the species now occurs in 14 provinces and regions [33]. During the summer, the temperature inside the *H. cunea* larval webs often exceeds 50 °C and can sometimes reach 60 °C. In spite of these high temperatures, the larvae are able to survive and develop [34]. In our previous studies, up to 50% of *H. cunea* larvae survived exposure to 40 °C for 62 h and even 43 °C for 14.5 h. [35]. The sHSP genes were considered to be related to the high temperature tolerance in invasive *H. cunea* [22,35]. For example, the recombinant *HCsHSP20.0* protein could protect malate dehydrogenase from thermal aggregation, and four sHSPs were sensitive to heat stress in the fourth-instar larvae of *H. cunea* [35]. These studies indicated that *H. cunea* has strong thermal tolerance. However, the molecular mechanisms of heat stress tolerance are unknown in *H. cunea*, and the function of sHSPs in other developmental stages remains poorly understood.

Based on previous transcriptomic data, we cloned and analyzed five novel sHSP genes from *H. cunea*. Quantitative Real-time PCR was used to detect the expression patterns in different developmental stages and various tissues suffering heat stress. The results will increase our knowledge of the sHSP family of *H. cunea* and advance further research on the mechanisms of *H. cunea* adaptation to high temperatures during its southward spread in China.

## 2. Results

### 2.1. Molecular Cloning and Sequence Analysis

Five sHSP genes (*HcHSP18.9*, *HcHSP20.1*, *HcHSP21.5*, *HcHSP21.4*, and *HcHSP29.8*) from *H. cunea* (GenBank accession numbers: OP964824, OP964825, OP964826, OP964827, and OP964828, respectively) were cloned. These five *HcHSP* genes contained ORFs of 501 bp, 528 bp, 576 bp, 564 bp, and 786 bp, which encoded 166, 175, 191, 187, and 261 amino acids, respectively. The predicted molecular weights of the *HcHSP* genes ranged from 18.9 to 29.8 kDa, the theoretical isoelectric points ranged from 5.79 to 6.38, and the instability indices ranged from 39.94 to 49.80. The instability index of *HcHSP18.9* was smaller than 40, but it was predicted to be stable (Table 1). The deduced amino acid sequences all contain the conserved alpha crystallin domain (ACD). Subcellular localization analysis indicated that HcHSP29.8 exists in the extracellular region and was predicted to have a signal peptide of 15 amino acids. The locations of the other four sHSPs were predicted to be in the cytoplasm (Table 1).

### 2.2. Multiple Sequence Alignments

**Figure 1:**
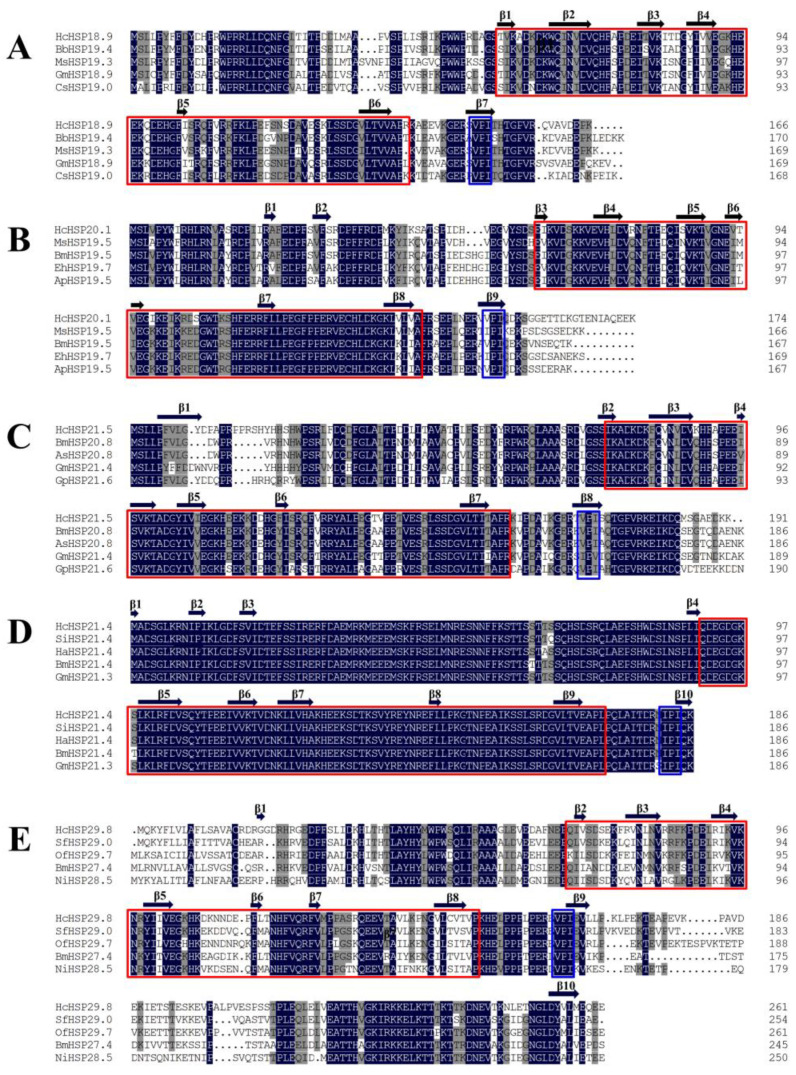
Alignment of the deduced amino acid sequences of five *HcHSPs* in *H. cunea*. (**A**) Amino acid sequence alignment of *HcHSP18.9* and four selected sHSPs from *Biston betularia* (ADO33017.1), *Mythimna separata* (ATN45241.1), *Grapholita molesta* (AKS40082.1), and *Chilo suppressalis* (QYR68970.1). (**B**) Amino acid sequence alignment of *HcHSP20.1* and four selected sHSPs from *Mythimna separata* (ATN45242.1), *Bombyx mori* (NP_001164470.2), *Eogystia hippophaecolus* (AYA93247.1), and *Antheraea pernyi* (APX61064.1). (**C**) Amino acid sequence alignment of *HcHSP21.5* and four selected sHSPs from *Bombyx mori* (NP_001091794.1), *Actias selene* (ALI87024.1), *Grapholita molesta* (AKS40075.1), and *Glyphodes pyloalis* (QGZ00461.1). (**D**) Amino acid sequence alignment of *HcHSP21.4* and four selected sHSPs from *Sesamia inferens* (AJA32863.1), *Helicoverpa armigera* (AGC39039.1), *Bombyx mori* (NP_001036985.1), and *Grapholita molesta* (AKS40074.1). (**E**) Amino acid sequence alignment of *HcHSP29.8* and four selected sHSPs from *Spodoptera frugiperda* (QLR06860.1), *Ostrinia furnacalis* (UTU55755.1), *Bombyx mandarina* (XP_028036235.1), and *Nymphalis io* (XP_050345805.1). Amino acids that were conserved in all sHSPs and over 75% of sHSPs are shaded in dark blue and grey, respectively. Secondary structures are indicated by arrows (β-sheets) and red rectangles (α-crystallin domain). The I/V-X-I/V motifs of the C terminal are indicated with blue boxes.

Using the five *HcHSPs* as query sequences, the basic local alignment search tool (BLAST) of NCBI (https://blast.ncbi.nlm.nih.gov/ (accessed on 2 April 2022)) was used to search for similar protein sequences. Then, several sequences from other insects with highly matching identities were selected, and the five *HcHSPs* were subjected to multiple sequence alignment with the selected genes. *HcHSP21.4* showed 99.47% similarity with homologous sHSPs from other insects, and the other four *HcHSPs* showed 71.19% to 83.79% similarity with other insect sHSPs (Figure 1, Table S1).

**Figure 2:**
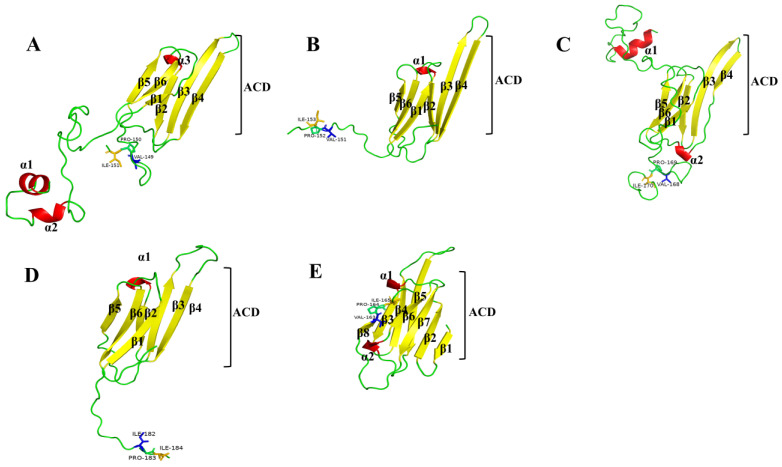
Predicted three-dimensional structures of five sHSPs in *H. cunea*: (**A**) *HcHSP18.9*; (**B**) *HcHSP20.1*; (**C**) *HcHSP21.5*; (**D**) *HcHSP21.4*; (**E**) *HcHSP29.8*. The red parts indicate the curly α-helix structure of the peptide chain, and the yellow parts indicate the β strands. The conserved residues corresponding to the I/V-X-I/V motif, including VALs or ILEs, PROs, and ILEs, are displayed as blue, green, and yellow sticks, respectively.

We predicted the secondary and three-dimensional protein structures of the five *HcHSP* genes. The amino acid sequences encoded by the five *HcHSPs* contained a typical α-crystallin domain, which consisted of approximately 80 amino acids, six to seven β-sheets, and the conserved motif I/V-X-I/V at the C-terminal (Figure 1). The predicted 3D structures showed that the α-crystallin domain exhibits a typical sandwich structure consisting of two β-sheets, each consisting of three and four antiparallel β-strands (Figure 2).

### 2.3. Phylogenetic Analysis

**Figure 3:**
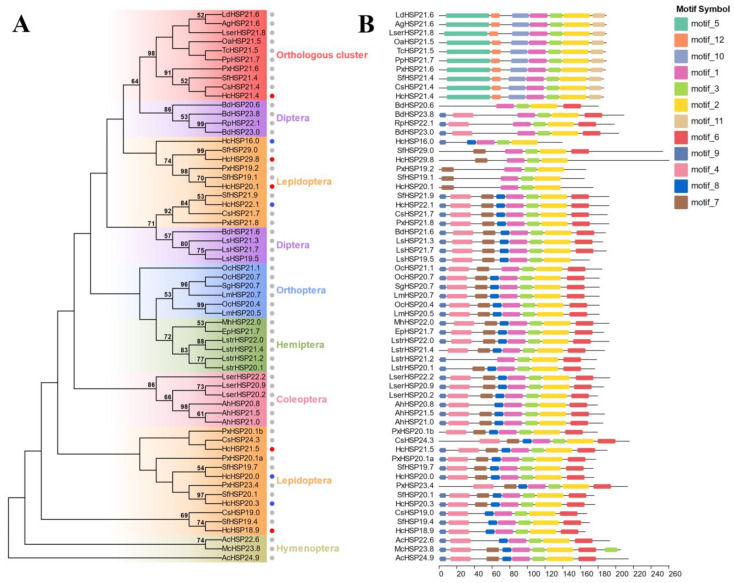
Phylogenetic analysis and motif analysis of sHSPs from *H. cunea* (the five *HcHSPs* and identified *HcHSPs*) and species in other orders (Lepidoptera, Hymenoptera, Coleoptera, Diptera, Orthoptera, and Hemiptera). (**A**) A phylogenetic tree was constructed using the neighbor-joining method with 1000 bootstrap replications. The five *HcHSPs*, the identified *HcHSPs*, and the sHSPs of other species in this study are labeled with red, blue, and gray circles, respectively. (**B**) MEME-based motif analysis. All motifs were identified using the complete sHSPs amino acid sequences. Motif lengths for each sHSP protein are shown proportionally. The sequence information of the species used in the construction of this phylogenetic tree and motif analysis is listed in the attached table.

To analyze the relationship between the sHSPs of *H. cunea* and the sHSPs of other insect species, sHSPs from different orders (Lepidoptera, Hymenoptera, Coleoptera, Diptera, Orthoptera, and Hemiptera) were compared with the sHSPs of *H. cunea* (including the four *HcHSPs* in this study and four previously published sHSPs) via phylogenetic tree analysis. None of the *HcHSPs* were clustered with each other, but they were clustered with sHSPs from other species of Lepidoptera. *HcHSP21.4* was alone on a branch, clustered with sHSPs from different species, and belonged to the orthologous HSP21.4-like protein group [27] (Figure 3A). Except for this cluster, sHSPs from insects of the same order were generally located on nearby branches. In addition, most *HcHSPs* were clustered on one branch with sHSPs of *Spodoptera frugiperda* (Figure 3A), suggesting that sHSPs of *H. cunea* are closely evolutionarily related to the sHSPs of Noctuidae.

Motif analysis showed that motifs 1, 2, and 3 occur in all HSP genes, which form a conserved ACD domain at the C-terminus. Motifs 5, 12, 10, and 11 were present only in orthologous sHSPs. Most of the species-specific sHSPs contained eight conserved motifs with similar motif distributions (Figure 3B).

### 2.4. Temporal and Spatial Expression Patterns of HcHSPs under Normal Conditions

Stage-specific expression patterns of the *H. cunea* sHSPs were determined at different developmental stages (eggs, second-instar larvae, fourth-instar larvae, sixth-instar larvae, pupae, female adults, and male adults) and in different tissues (the gut, Malpighian tubules, silk glands, hemolymph, fat bodies, and the cuticle) via quantitative RT-qPCR reactions.

**Figure 4:**
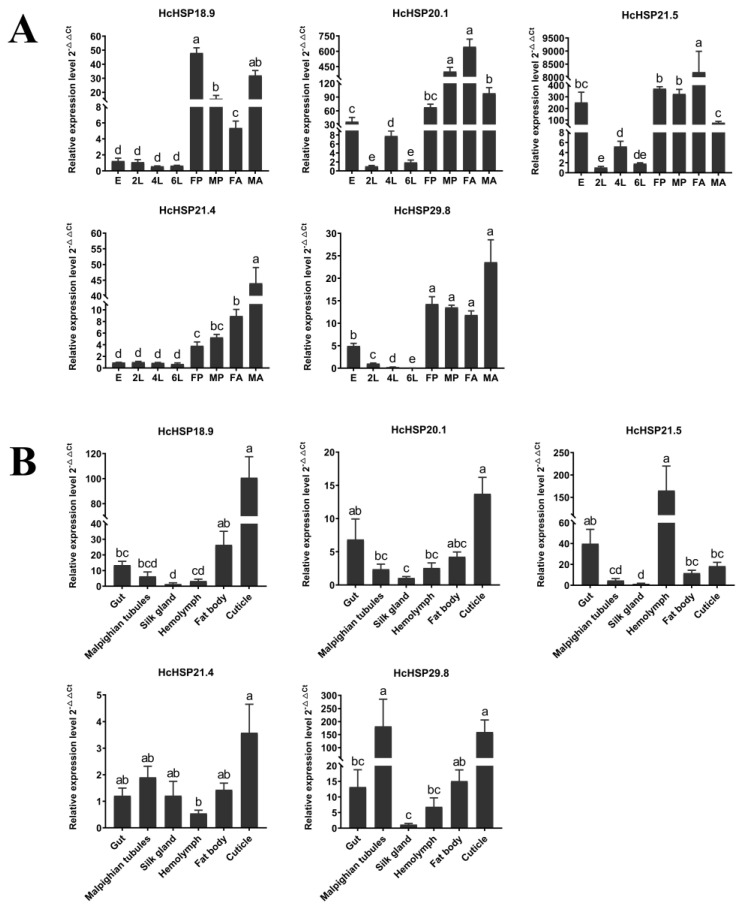
Relative mRNA expression levels of five *HcHSPs* in different developmental stages and various 4th-instar larvae tissues of *H. cunea*. (**A**) The different developmental stages were as follows: E: eggs; L2: 2nd-instar larvae; L4: 4th-instar larvae; L6: 6th-instar larvae; FP: female pupae; MP: male pupae; FAs: female adults; MAs: male adults. The relative expression levels of the *HcHSPs* were determined via comparison with the 2nd-instar larvae. (**B**) The various tissues from 4th-instar larvae were as follows: gut, Malpighian tubules, silk gland, hemolymph, fat body, and cuticle. The relative expression levels of the *HcHSPs* were determined via comparison with silk glands. The values are expressed as means ± SE. Different lowercase letters indicate statistically significant differences (*p* < 0.05).

In the temporal expression patterns analysis, it was observed that most *HcHSPs* were enriched in the pupal and adult stages, with the lowest expression in the sixth-instar larvae. *HcHSP18.9* exhibited the highest expression in the female pupae and male adults, while *HcHSP20.1* was highly expressed in male pupae and female adults. *HcHSP21.5* was highest in female adults and was also highly expressed in eggs and pupae. *HcHSP21.4* and *HcHSP29.8* exhibited high expression in male adults (Figure 4A).

In the spatial expression pattern analysis, the five HcHSP genes were expressed in larval tissues. However, there were significant differences in the expression patterns of the different sHSPs among the tested tissues. *HcHSP21.4* was more evenly distributed in all tissues, where it showed high expression levels (Figure 4B). The expression levels of *HcHSP18.9*, *HcHSP20.1*, and *HcHSP29.8* were highest in the cuticle tissues, followed by the gut, fat body, and Malpighian tubules. The expression levels of *HcHSP21.5* was highest in the hemolymph tissues. The relative expression of most *HcHSP* genes was lowest in the silk glands (Figure 4B).

### 2.5. Heat-Induced Expression of HcHSPs on the Temporal and Spatial Scales

#### 2.5.1. Heat-Induced Expression Profiles of HcHSPs in Different Developmental Stages

**Figure 5:**
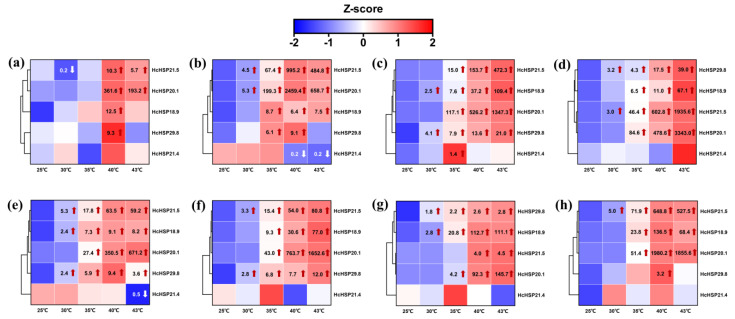
Temporal expression of *HcHSPs* under different high temperature treatments. Only the expressions with significant differences are labeled in the figure. Red arrows indicate significantly up-regulated expression, while white arrows indicate significantly down-regulated expression, and numbers indicate the fold-change values of gene up-regulation. Quantitative data of gene expression levels were Z-score-normalized. (**a**) Eggs; (**b**) 2nd-instar larvae; (**c**) 4th-instar larvae; (**d**) 6th-instar larvae; (**e**) female pupae; (**f**) male pupae; (**g**) female adults; (**h**) male adults. Note: A detailed expression and significance analysis is shown in Table S6.

After heat treatment, the expression levels of the five *HcHSPs* in the different developmental stages were determined. The expression of *HcHSP21.4* did not change significantly under heat stress and was only slightly down-regulated in some developmental stages. The expression levels of *HcHSPs* increased significantly, except for *HcHSP21.4*, and the relative expression of most *HcHSPs* increased with an increasing treatment temperature. In the eggs, *HcHSPs* started to be transcriptionally up-regulated at 40 °C (Figure 5). In the larvae, pupae, and adults, *HcHSP* expression was up-regulated immediately under the 30 °C and 35 °C treatments. The induction peak occurred at 43 °C (Figure 5).

Under the high temperature treatment, the expression patterns of different *HcHSPs* varied among the different developmental stages of *H. cunea*. *HcHSP20.1* and *HcHSP21.5* showed the strongest responses to high temperature. *HcHSP20.1* was heavily transcribed in all developmental stages under high-temperature stress. The induction peaks of *HcHSP20.1* were 2459.4-, 1347.3-, and 3343.0-fold greater in the second-, fourth-, and sixth-instar larvae; 671.2- and 1652.6-fold greater in the female and male pupae; and 145.7- and 1980.2-fold greater in the male and female adults. In contrast, the expression levels of *HcHSP21.5* were high at the larval stage and in the female adults; 995.2-, 472.3-, and 1935.6-fold higher than the control in the second-, fourth-, and sixth-instar larvae, respectively; and 678.79-fold higher than the control in the male adults. There was a weaker response in the other stages. The expression patterns of *HcHSPs* differed among the sexes in the pupae and adults. *HcHSP18.9* showed greater up-regulation in male pupae, while *HcHSP20.1* and *HcHSP21.5* were most up-regulated in male adults (Figure 5).

#### 2.5.2. Heat-Induced Expression Profiles of HcHSPs in Various Tissues

**Figure 6:**
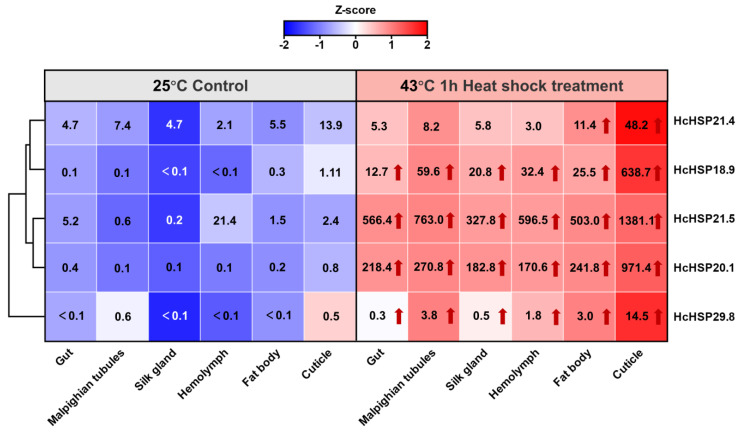
Tissue-specific gene relative expression of *HcHSPs* in *H. cunea* 4th-instar larvae under normal temperature (25 °C) and after high temperature (43 °C) treatment. Red cells indicate high expression, while blue cells indicate low expression. Red arrows in the cells indicate significant up-regulation, and numbers indicate the relative expression of *HcHSPs* genes. Quantitative data of gene expression levels were Z-score-normalized. Note: A detailed significance analysis is shown in Table S7.

After the high-temperature treatment (43 °C for 1 h), the expression levels of *HcHSP18.9*, *HcHSP20.1*, *HcHSP21.5*, and *HcHSP29.8* were significantly up-regulated in all the tested tissues. In contrast, *HcHSP21.4* expression was only slightly up-regulated in some tissues (fat body and cuticle). Under heat stress, the expression levels of *HcHSP18.9*, *HcHSP20.1*, *HcHSP21.5*, and *HcHSP29.8* were highest in the cuticle, followed by the Malpighian tubules and fat body, with the lowest levels observed in the silk gland and gut. In addition, the relative expression of most sHSP genes was lowest in the silk glands but up-regulated to the greatest extent after heat stress. These results suggest that, during heat stress, the *HcHSP* genes respond differently in different tissues (Figure 6).

## 3. Discussion

Under high-temperature-stress conditions, sHSPs act as a molecular chaperone that prevents the irreversible aggregation of misfolded proteins [11,12]. Insects possess several types of sHSPs, which differ in structure and function [5,36,37]. We identified and cloned five *sHSP* genes of *H. cunea*. Based on multiple sequence comparisons, phylogenetic analysis, and motif prediction, we identified *HcHSP21.4* as belonging to the orthologous protein group. Sequence analysis indicated that the five *H. cunea* sHSPs had high similarity with the sHSPs of other Lepidoptera species. The deduced amino acid sequences of the *HcHSPs* contained a conserved ACD at the C-terminus, which is a β-sandwich structure consisting of six to seven antiparallel β-strands [12]. This structural homogeneity aligns with the shared characteristics observed in small heat shock proteins (sHSPs) across diverse insect species [37,38]. Furthermore, it is noteworthy that a conserved I/V-X-I/V motif has been unequivocally identified within the C-terminal region of all five *HcHSPs*. This particular motif serves a pivotal role as an anchor point during the process of oligomerization, a phenomenon commonly associated with sHSPs. It is involved in the stabilization of sHSP assembly and contributes to structural diversity in α-crystallins [11,12]. However, the amino acid lengths and sequences of the N-terminal extensions are highly variable, possibly reflecting the monophyletic origin of the α-crystallin domain and the independent evolution of the flanking regions. This situation increased variability in specific small parts of the protein and facilitated the functional and structural differentiation of sHSPs [39]. These results suggest that the high variation of sHSP sequences contrasts markedly with the conserved patterns of the other HSP families [12]. In addition, diversity in the subcellular localization and signal peptide sequences of *HcHSPs* was also observed. *HcHSP18.9*, *HcHSP20.1*, *HcHSP21.5*, and *HcHSP21.4* were predicted to be in the cytoplasm, confirming the involvement of sHSPs in important intracellular physiological processes. *HcHSP29.8* was predicted to be in the extracellular region and to contain a signal peptide. This suggests that it functions extracellularly as a secretory protein after transmembrane transport [40,41].

The sHSPs of Insects can be divided into two categories (orthologous clusters and species-specific clusters) [27] according to the clustering methods used to construct the phylogenetic trees [42,43]. Phylogenetic analysis revealed that *HcHSP21.4* is clustered on a branch that belongs to an orthologous cluster of HSP21.4-like proteins consisting of one sHSP from each insect species [44,45]. Most insect sHSPs are species-specific [27], as demonstrated by the other four sHSPs in this study, and this clustering method shows the evolutionary pattern of lineage-specific expansion. Furthermore, the species’ sHSPs do not cluster with each other but instead cluster with sHSPs from other insects of the same order (Figure 3). This is consistent with research on *Plutella xylostella*, *Chilo suppressalis*, and *Spodoptera litura* [5,42,43]. The interspecific relationships of *H. cunea* sHSPs appear to be more akin to intraspecific relationships, suggesting that sHSP genes might have duplicated early in insect evolution [44]. These results further validate the accuracy of the sequence and motif analysis in this study. Notably, the phylogenetic relationship between *HcHSPs* and the sHSPs found in Noctuidae exhibits a remarkable degree of similarity. It has also been shown that Erebidae and Noctuidae belong to the superfamily Noctuoidae [46], with a similar taxonomic status. These results demonstrate that the amino acid sequences of insect sHSPs are less conservative, with only closely related species showing high similarity [12].

Variation in the expression levels of sHSPs in the developmental stage is widely observed in insects. In this study, we found the expression of *HcHSPs* reached maximum levels in the pupal and adult stages. Similar phenomena have been observed in other insects. For example, most of the sHSPs of *P. xylostella* [5], and *HSP19.7*, *HSP20.0*, and *HSP20.8* of *S. litura* [43], were expressed at the highest levels in pupae and adults. This suggests that HSPs may be involved in specific physiological events in insect pupae and adults. The pupal stage is a critical period of metamorphosis, and it is also a period of high basal metabolism, which may induce sHSPs expression [20]. sHSPs may also act as chaperone proteins for promoting tissue and organ degradation and remodeling during the pupal stage [7]. *Hyphantria cunea* demonstrates a remarkable reproductive capacity, characterized by an average fecundity of 800–900 eggs per female and a maximum potential of up to 2000 eggs [47]. High expression of sHSPs may be involved in the reproductive events of adults, including the development of the reproductive system and the formation of germ cells [48].

The up-regulation of HSP expression levels contributes to increased heat tolerance [49]. As members of the HSP family, sHSPs play an essential role in resistance to heat stress. We found that the relative expression levels of *HcHSPs*, except for *HcHSP21.4*, were significantly up-regulated under heat stress and continued to increase with the increase in temperature stress. In previous studies, four of the six identified sHSPs were sensitive to high temperatures [35]. These results indicate that *H. cunea* has strong heat tolerance, which may enhance its survival as it spreads to the warmer southern areas of China.

Previous studies have indicated that sHSPs play an important role in the regulation of insect growth and development [20,50]. Expression patterns differed at different developmental stages of *H. cunea*. In general, the transcription of *HcHSP* genes was more sensitive to high temperature. In the developmental stages other than eggs, the transcription of *HcHSPs* was immediately up-regulated at 30 or 35 °C, and expression continued to be up-regulated with an increasing temperature. Heat stress constitutes an exogenous stressor affecting cellular functions, and the prompt induction of Heat Shock Proteins (HSPs) serves to safeguard cellular proteins and uphold an organism’s physiological homeostasis [51]. In contrast, in this study, the expression level of *HcHSPs* began to be up-regulated when the eggs were exposed to a heat stress treatment of 40 °C. The sexually dimorphic *Ericerus pela* has a thick wax layer on the body surface that is insensitive to high temperatures [52]. Similarly, the wax layer structure of the outer eggshells of *H. cunea* eggs [53] may form a protective barrier that makes the eggs less sensitive to high temperatures.

Our results also showed that *HcHSPs* respond differently to high temperatures at different developmental stages and that the expression patterns varied with the growth stage. Under high-temperature stress, *HcHSP20.1* was highly expressed throughout the life cycle of *H. cunea*, while *HcHSP21.5* was strongly expressed in larvae and male adults. Multiple and distinct sHSP response networks may exist throughout a life cycle, with *H. cunea* regulating the expression of different sHSPs at different developmental stages in response to high temperatures. These results indicate that *HcHSPs* play important roles in the heat resistance of *H. cunea* at different developmental stages. *HcHSPs* were more highly expressed in male adults than female adults. A similar phenomenon was observed in *Frankliniella occidentalis* [15]. This is probably because female adults of *Hcunea* are larger and more thermotolerant than male adults.

The expression pattern of sHSPs in insects is tissue-specific. For example, in male adults of *S. frugiperda*, the expression levels of *SfsHsp20.1* and *SfsHsp19.3* were highest in the abdomen, while those of *SfsHsp21.3*, *SfsHsp20*, and *SfsHsp29* were highest in the thorax, head, and compound eyes, respectively [54]. In *Lasioderma serricorne*, *LsHSP19.4* and *LsHSP20.3* were most highly expressed in the fat body, and *LsHSP20.2* was most highly expressed in the gut [38]. In *H. cunea*, five *HcHSPs* were widely distributed in the larval tissues. The expressions of *HcHSPs* were highest in the cuticle, similar to the expression of *HSP19.22*, *HSP19.23*, and *HSP20.09* in *P. xylostella* [5] and *BmHSP22.6* and *BmHSP19.1* in *B. mori* [27]. Under normal conditions, *HcHSPs* were highly expressed in the cuticle, which indicates their involvement in larval growth and development. In addition, the expression of *HcHSPs* remained highest in the epidermis after heat stress and was greatly up-regulated. The cuticle is an immunocompetent tissue, and sHSPs may play a role in the immune defense mechanism of *H. cunea* against external heat stress [27]. However, the reasons for the high expression of sHSPs in the tested tissues are unclear, and further studies are needed to elucidate their exact role in *H. cunea*.

The similarity between the amino acid sequences encoded by *HcHSP21.4* and HSP21.4-like proteins of other lepidopteran species (*Sesamia inferens*, *Helicoverpa armigera*, *B. mori*, and *Grapholita molesta*) was as high as 99.47%. This shows high conservation in the entire sequence. The conserved α crystal domain is more conserved than the species-specific sHSPs, and this is a major structural feature of genes in the orthologous cluster [27,42,43]. As a constitutive gene, *HcHSP21.4* was insensitive to heat stress and maintained a constant expression level in different tissues and at different developmental stages (Figure 4, Figure 5 and Figure 6). The same findings were observed for the *HSP21.4* gene of *S. litura* [43], the *HSP21.8* gene of *P. xylostella* [5], and the *HSP21.8b* gene of *T. castaneum* [55]. These genes are considered housekeeping genes, and *HcHSP21.4* may play an important role in maintaining the basal metabolism of tissues and the growth and development of insects [27]. On the other hand, the overexpression of sHSPs may have harmful effects on insects. For example, the overexpression of sHSP negatively affects the fecundity of *Liriomyza trifolii* females [56]. Therefore, orthologous sHSPs are slightly expressed or not expressed under stress conditions to maintain the normal life activities of insects [25,55].

## 4. Materials and Methods

### 4.1. Insects

Fourth-instar larvae of *H. cunea* were collected from *Populus deltoides* in Huaian City, Jiangsu Province, China (33.62° N, 119.03° E), and reared on fresh *P. deltoides* leaves in an incubator at 25 ± 1 °C with 60 ± 5% humidity and a 16:8 h (L:D) photoperiod. Leaves were replaced daily until the larvae pupated. Newly emerged adults were placed in screen cages (40 × 50 × 30 cm) for mating and oviposition on fresh *P. deltoides* leaves. The second-generation *H. cunea* subjects were used in the subsequent experiments.

### 4.2. Sample Preparation

Our previous investigations revealed that the Ltim50 values for *H. cunea* 4th-instar larvae were 62.0 h at 40 °C, 14.5 h at 43 °C, and even 2.3 h at 45 °C [35]. To consider the differences in the thermotolerance of *H. cunea* at different developmental stages, we set the treatment duration to one hour and used 43 °C as the upper temperature limit. Two-day-old eggs (*n* = 300), two-day-old 2^nd^-instar larvae (*n* = 50), two-day-old 4^th^-instar larvae (*n* = 10), two-day-old 6^th^-instar larvae (*n* = 10), two-day-old female and male pupae (*n* = 10), and two-day-old female and male adults (*n* = 10) were individually placed in plastic boxes (14 × 8 × 6 cm) and exposed to different temperature treatments (30, 35, 40, and 43 °C) for 60 min using a temperature gradient incubator (60 ± 5% RH, 16L:8D). 

After treatment, the vigorous individuals from each treatment were randomly sampled for subsequent RNA extraction and qPCR experiments. Since the embryos in the eggs did not feed or move, it was not possible to ensure their health. Each replicate was divided into two groups at the time of treatment, namely, one for freezing for subsequent experiments and another group that continued to be reared at room temperature after treatment until the eggs hatched, and the frozen samples were considered as valid samples if the eggs hatched. Each replicate consisted of 100 eggs, ten 2nd-instar larvae, one 4th-instar larva, one 6th-instar larva, one female pupa, one male pupa, one female adult, and one male adult, respectively, and three replicates per treatment were used in this experiment. Various tissues (including guts, Malpighian tubules, silk glands, hemolymph, fat bodies, and cuticles) of 4th-instar larvae treated at 43 °C were dissected and sampled for RNA extraction and qPCR experiments. The tissues from five larvae were pooled to represent a biological replicate. All samples were immediately frozen in liquid nitrogen and kept at −80 °C until tested. In all experiments, a negative control group of *H. cunea* at the same stage was maintained at 25 °C for 60 min. Three biological replicates were employed for each treatment.

### 4.3. RNA Extraction and cDNA Synthesis

Total RNA was extracted using Trizol reagent (TIANGEN, Beijing, China) according to manufacturer’s instructions. The quality and purity of RNA after DNAase treatment were determined using NanoDrop 2000C spectrophotometer (Thermo Scientific, Waltham, MA, USA) and 1% gel electrophoresis. Finally, cDNA was synthesized from 1 μg of RNA of each sample using the Hiscript First Strand cDNA Synthesis Kit (Vazyme, Nanjing, China) and used for PCR and RT-qPCR.

### 4.4. HcHSPs Sequence Cloning

The original sequences of sHSP genes were obtained from *H. cunea* transcriptome data (unpublished by our laboratory). Primers for PCR amplification of sHSP Open Reading Frame (ORF) were designed using an online tool (https://blast.ncbi.nlm.nih.gov/Blast.cgi (accessed on 25 March 2022)) and are listed in Table 1. The PCR program consisted of 30 cycles, 98 °C for 10 s, 55 °C for 30 s, and 72 °C for 1 min. PCR products were detected on 1% agarose gel and extracted with a DNA purification kit (TIANGEN, Beijing, China). The PCR products were cloned into a T/A vector and transformed into DH5α competent cells (Vazyme, Nanjing, China). After 14 h, positive clones were selected and sequenced by Sangon, Shanghai, China.

### 4.5. Bioinformatics and Phylogenetic Analyses

The conserved domains of sHSP were predicted using the online software CD-Search (https://www.ncbi.nlm.nih.gov/structure/cdd/wrpsb.cgi (accessed on 3 December 2022)). The molecular weights, isoelectric points, and hydrophobicity values were predicted using the ExPASy tool (https://web.expasy.org/ (accessed on 3 December 2022)). Signal peptides were identified using SignalP 4.1 (https://services.healthtech.dtu.dk/service.php?SignalP-4.1 (accessed on 3 December 2022)). The subcellular locations were predicted using the WoLF PSORT tool (https://wolfpsort.hgc.jp/ (accessed on 3 December 2022)). Multiple sequence alignments were generated using the DNAMAN 8.0 sequence analysis software (Lynnon Biosoft, San Ramon, CA, USA). The secondary structure of sHSP proteins was analyzed with SOPMA (http://npsa-pbil.ibcp.fr/cgi-bin/npsa_automat.pl?page=npsa_sopma.html (accessed on 5 December 2022)). The online protein structure prediction tool SWISS-MODEL (https://swissmodel.expasy.org/ (accessed on 5 December 2022)) was used to predict the tertiary structures of the sHSP genes, and PyMOL 2.5 (https://pymol.org/2/ (accessed on 22 February 2023)) was used to visualize the proteins’ 3D structures. A phylogenetic tree was constructed based on the deduced amino sequences of sHSPs from *H. cunea* and insects from different orders in the NCBI database. This was performed using MEGA 11 software and the neighbor-joining method with 1000 iterations; values lower than 50% are not shown. The online tool ChiPlot (https://www.chiplot.online/ (accessed on 10 May 2023)) was used to visualize the phylogenetic tree. The MEME online program (http://meme.nbcr.net/meme/intro.html (accessed on 10 May 2023)) was used for motif analysis.

### 4.6. RT-qPCR

The Applied Biosystem 7500 System (Applied Biosystems, Foster City, CA, USA) was used to perform qPCR in a 20 μL volume containing 10 μL of Hieff UNICON^®^ qPCR SYBR Green Master Mix (YEASEN, Shanghai, China); 0.4 µL of (10 µM) of each gene-specific primer; 2 µL of cDNA; and 7.2 µL of nuclease-free water. All primers used for qPCR were designed online using Primer-BLAST (https://www.ncbi.nlm.nih.gov/tools/primer-blast/ (accessed on 21 May 2022)), available on the NCBI website (Table 2). The qPCR procedure was as follows: 95 °C for 5 min, 40 cycles of 95 °C for 10 s, and 60 °C for 40 s. We then conducted a melting curve analysis for continuous fluorescence monitoring to ensure the specificity and consistency of the products amplified. The EF1α gene was used as an internal reference gene [57]. The relative expression levels of sHSP genes at different developmental stages and various tissues under normal conditions were calculated using the 2^−ΔΔCT^method [58]; the relative expression levels of sHSP genes in tissues after heat shock treatment were calculated using the 2^−ΔCT^method [58].

### 4.7. Data Analysis

The mRNA levels of sHSPs in the same tissues under the control and heat treatments were compared using independent samples *t*-tests. Statistically significant differences in other quantitative data were analyzed using one-way ANOVA followed by Tukey’s HSD test. Quantitative data of gene heat-induced expression levels were Z-score-normalized before plotting the heatmaps. Each sHSP gene was normalized at different developmental stages and in various tissues, respectively. All values reported in the text are means ± standard error (SE). The data were log_2_-transformed, and the homogeneity of the data was tested before conducting ANOVA. *p* < 0.05 indicated a statistically significant difference. All statistical analyses were conducted using SPSS 21.0 software (IBM SPSS Statistics, Chicago, IL, USA), and plots were generated with GraphPad Prism 8.0 (GraphPad Software, La Jolla, CA, USA) and online tool ChiPlot (https://www.chiplot.online/ (accessed on 27 July 2023)). Flowcharts were generated using BioRender.com.

## 5. Conclusions

We identified five new sHSPs in *H. cunea* and analyzed their transcriptional expression profiles at different developmental stages and in different tissues under heat stress. All of these putative amino acid sequences contain the conserved ACDs of sHSPs, which are diverse in terms of subcellular localization and signal peptide sequence prediction. *HcHSPs* were expressed at the highest levels in the pupal and adult stages. They may be involved in metamorphosis in the pupal stage and reproductive events in the adult stage. The relative expression levels of *HcHSPs* were significantly up-regulated under heat stress and continued to increase with the increase in temperature. Concerning eggs, the wax layer structures of the eggshells may help reduce sensitivity to high temperatures. The high expression of *HcHSPs* in the cuticle may be related to the immune defense function. The orthologous protein *HcHSP21.4* is not sensitive to high temperatures but may be involved in the regulation of normal physiological metabolism. In conclusion, the results suggest that *HcHSPs* play an important role in metamorphosis and reproduction as well as in the resistance to heat stress in *H. cunea*. These functions will help *H. cunea* adapt to the higher temperatures of southern China. This study provides a better understanding of the sHSPs family in *H. cunea*. However, the exact role of the heat resistance mechanism of *HcHSPs* requires further study.

## Figures and Tables

**Table 1:** Characteristics of the mRNAs of sHSPs of *H. cunea*.

Gene Name	GenBank Accession Number	ORF (bp)	Protein Length (aa)	Molecular Weight (kDa)	Isoelectric Point (IP)	Instability Index (II)	Signal Peptide (AA)	Subcellular Location
HcHSP18.9	OP964824	501	166	18.9	5.96	39.94	No	Cytoplasmic
HcHSP20.1	OP964825	528	175	20.1	6.00	49.62	No	Cytoplasmic
HcHSP21.5	OP964826	576	191	21.5	6.38	49.80	No	Cytoplasmic
HcHSP21.4	OP964827	564	187	21.4	5.79	48.88	No	Cytoplasmic
HcHSP29.8	OP964828	786	261	29.8	5.84	46.27	15	Extracellular

**Table 2:** Primers used in this study.

Application	Primer Name	Forward Primer (5′-3′)	Reverse Primer (5′-3′)
PCR	*HcHSP18.9*	ATGTCTCTTTTGCCATACTTCT	CTATTTTGGTTCATCAACAGCAACC
	*HcHSP20.1*	ATGTCATTGGTGCCGTATTGG	CTAAGCTTTTTCTTCCTGTGCT
	*HcHSP21.5*	ATGTCTCTGCTACCATTTGTTTTGG	TTACTTCTTATCTTCAGCGCCG
	*HcHSP21.4*	ATGGCTGATAGTGGTCTGAAGA	TCAGTGCTTCTGGATAGGGA
	*HcHSP29.8*	ATGCAGAAATATTTCTTAGTACTCGCA	TTACTCTTCTTGTTCCATTAGTACA
qPCR	*HcHSP18.9*	AAGCTGTCTTCGGATGGTGT	GCCTCACGGGTCCTGTATG
	*HcHSP20.1*	CAGTGGCTGGACCAAGAGTC	CCGATCTAAACGCCACCAGA
	*HcHSP21.5*	GAATCCCGGCTTTCATCCGA	CCTGTCTGCGAAATAGGCAC
	*HcHSP21.4*	ACATCGTCACAACACAGCGA	GCTTGAGTGACTTGCCGTCT
	*HcHSP29.8*	GAGGCGAAGACCCATTCTCC	CAGTTGACTCCAAGGCCACA
	*EF1α*	TTATCGTCGCTGCTGGTACA	GAGTGTGAAAGCGAGCAGAG

## Data Availability

The data presented in this study are available on request from the corresponding author.

## Supplementary Materials

The following supporting information can be downloaded at: https://www.mdpi.com/article/10.3390/ijms242015176/s1.

## References

[1] Lindquist S., Craig E.A. (1988). The Heat-Shock Proteins. Annu. Rev. Genet..

[2] Ritossa F. (1962). A New Puffing Pattern Induced by Temperature Shock and DNP in Drosophila. Experientia.

[3] Zagoriti Z., Georgitsi M., Giannakopoulou O., Ntellos F., Tzartos S.J., Patrinos G.P., Poulas K. (2012). Genetics of Myasthenia Gravis: A Case-Control Association Study in the Hellenic Population. J. Immunol. Res..

[4] Parsell D.A., Lindquist S. (1993). The Function of Heat-Shock Proteins in Stress Tolerance: Degradation and Reactivation of Damaged Proteins. Annu. Rev. Genet..

[5] Chen X.E., Zhang Y.L. (2015). Identification of Multiple Small Heat-Shock Protein Genes in *Plutella Xylostella* (L.) and Their Expression Profiles in Response to Abiotic Stresses. Cell Stress Chaperones.

[6] Miano F.N., Jiang T., Zhang J., Zhang W.-N., Peng Y., Xiao H.-J. (2022). Identification and Up-Regulation of Three Small Heat Shock Proteins in Summer and Winter Diapause in Response to Temperature Stress in *Pieris Melete*. Int. J. Biol. Macromol..

[7] Feder M.E., Hofmann G.E. (1999). Heat-Shock Proteins, Molecular Chaperones, and the Stress Response: Evolutionary and Ecological Physiology. Annu. Rev. Physiol..

[8] King A.M., MacRae T.H. (2015). Insect Heat Shock Proteins During Stress and Diapause. Annu. Rev. Entomol..

[9] Haslbeck M. (2002). SHsps and Their Role in the Chaperone Network. Cell. Mol. Life Sci..

[10] Franck E., Madsen O., van Rheede T., Ricard G., Huynen M.A., de Jong W.W. (2004). Evolutionary Diversity of Vertebrate Small Heat Shock Proteins. J. Mol. Evol..

[11] Poulain P., Gelly J.C., Flatters D. (2010). Detection and Architecture of Small Heat Shock Protein Monomers. PLoS ONE.

[12] Basha E., O’Neill H., Vierling E. (2012). Small Heat Shock Proteins and α-Crystallins: Dynamic Proteins with Flexible Functions. Trends Biochem. Sci..

[13] Basha E., Friedrich K.L., Vierling E. (2006). The N-Terminal Arm of Small Heat Shock Proteins Is Important for Both Chaperone Activity and Substrate Specificity. J. Biol. Chem..

[14] Horwitz J. (1992). Alpha-Crystallin Can Function as a Molecular Chaperone. Proc. Natl. Acad. Sci. USA.

[15] Yuan J.W., Song H.X., Chang Y.W., Yang F., Xie H.F., Gong W.R., Du Y.Z. (2022). Identification, Expression Analysis and Functional Verification of Two Genes Encoding Small Heat Shock Proteins in the Western Flower Thrips, *Frankliniella Occidentalis* (Pergande). Int. J. Biol. Macromol..

[16] Huang L.H., Wang C.Z., Kang L. (2009). Cloning and Expression of Five Heat Shock Protein Genes in Relation to Cold Hardening and Development in the Leafminer, *Liriomyza Sativa*. J. Insect Physiol..

[17] Sonoda S., Ashfaq M., Tsumuki H. (2006). Cloning and Nucleonde Sequencing of Three Heat Shock Protein Genes (Hsp90, Hsc70, and Hsp19.5) from the Diamondback Moth, *Plutella Xylostella* (L.) and Their Expression in Relation to Developmental Stage and Temperature. Arch. Insect Biochem. Physiol..

[18] Takahashi K.H., Rako L., Takano-Shimizu T., Hoffmann A.A., Lee S.F. (2010). Effects of Small Hsp genes on Developmental Stability and Microenvironmental Canalization. BMC Evol. Biol..

[19] Li H., Hao D.J., Xu T., Dai L.L. (2022). The Effects of Heat Stress on Herbivorous Insects: An Overview and Future Direction. J. Nanjing For. Univ. Sci. Ed..

[20] Dong C.L., Zhu F., Lu M.X., Du Y.Z. (2021). Characterization and Functional Analysis of Cshsp19.0 Encoding a Small Heat Shock Protein in *Chilo Suppressalis* (Walker). Int. J. Biol. Macromol..

[21] Garczynski S.F., Unruh T.R., Guedot C., Neven L.G. (2011). Characterization of Three Transcripts Encoding Small Heat Shock Proteins Expressed in the Codling Moth, *Cydia Pomonella* (Lepidoptera: Tortricidae). Insect Sci..

[22] Zheng H.Y., Qin P.H., Yang K., Liu T.X., Zhang Y.J., Chu D. (2022). Genome-Wide Identification and Analysis of the Heat-Shock Protein Gene Superfamily in *Bemisia Tabaci* and Expression Pattern Analysis under Heat Shock. Insects.

[23] Liu Q.N., Zhu B.J., Dai L.S., Fu W.W., Lin K.Z., Liu C.L. (2013). Overexpression of Small Heat Shock Protein 21 Protects the Chinese Oak Silkworm *Antheraea Pernyi* against Thermal Stress. J. Insect Physiol..

[24] Chu J., Jiang D.L., Ya M.W., Li Y.J.C., Wang J., Wu F.A., Sheng S. (2020). Identifications, Characteristics, and Expression Patterns of Small Heat Shock Protein Genes in a Major Mulberry Pest, *Glyphodes Pyloalis* (Lepidoptera: Pyralidae). J. Insect Sci..

[25] Xie J., Peng G.F., Hu X.X., Gu S.S., Bi J.X., Wei L.T., Tang J., Song X.W., Feng F., Li B. (2020). Functional Analysis of a Novel Orthologous Small Heat Shock Protein (Shsp) Hsp21.8a and Seven Species-Specific Shsps in *Tribolium Castaneum*. Genomics.

[26] Quan G., Duan J., Ladd T., Krell P.J. (2018). Identification and Expression Analysis of Multiple Small Heat Shock Protein Genes in Spruce Budworm, *Choristoneura Fumiferana* (L.). Cell Stress Chaperones.

[27] Li Z.W., Li X., Yu Q.Y., Xiang Z.H., Kishino H., Zhang Z. (2009). The Small Heat Shock Protein (SHSP) Genes in the Silkworm, *Bombyx Mori*, and Comparative Analysis with Other Insect SHSP Genes. BMC Evol. Biol..

[28] Zhao X., Geng Y., Hu T., Xie C., Xu W., Zuo Z., Xue M., Hao D. (2023). Ecological Strategies of *Hyphantria Cunea* (Lepidoptera: Arctiidae) Response to Different Larval Densities. Front. Ecol. Evol..

[29] Zhao X., Geng Y., Hu T., Li W., Liang Y., Hao D. (2023). Comparing the Performance of *Hyphantria Cunea* (Lepidoptera: Arctiidae) on Artificial and Natural Diets: Feasibility of Mass-Rearing on Artificial Diets. J. Econ. Entomol..

[30] Edosa T.T., Jo Y.H., Keshavarz M., Anh Y.S., Noh M.Y., Han Y.S. (2019). Current Status of the Management of Fall Webworm, *Hyphantria Cunea*: Towards the Integrated Pest Management Development. J. Appl. Entomol..

[31] Sullivan G.T., Karaca I., Ozman-Sullivan S.K., Kara K. (2012). Tachinid (Diptera: Tachinidae) Parasitoids of Overwintered *Hyphantria Cunea* (Drury) (Lepidoptera: Arctiidae) Pupae in Hazelnut Plantations in Samsun Province, Turkey. J. Entomol. Res. Soc..

[32] Zhao L.Q., Wang X.M., Liu Z., Torson A.S. (2022). Energy Consumption and Cold Hardiness of Diapausing Fall Webworm Pupae. Insects.

[33] Li Z., Yin H., Li Y., Wang Y., Yu W., Feng B., Zhang S. (2023). *Hyphantria Cunea* (Drury) Showed a Stronger Oviposition Preference for Native Plants after Invading the Subtropical Region of China. Agronomy.

[34] Rehnberg B.G. (2006). Temperature Profiles inside Webs of the Fall Webworm, *Hyphantria Cunea* (Lepidoptera: Arctiidae): Influence of Weather, Compass Orientation, and Time of Day. J. Therm. Biol..

[35] Li H., Tao R., Qiao H., Zhao X.D., Li S.Y., Dai L.L., Hao D.J. (2022). Functional Analysis of Small Heat Shock Proteins Providing Evidence of Temperature Tolerance in *Hyphantria Cunea*. J. Appl. Entomol..

[36] Haslbeck M., Kastenmüller A., Buchner J., Weinkauf S., Braun N. (2008). Structural Dynamics of Archaeal Small Heat Shock Proteins. J. Mol. Biol..

[37] Wang L., Zhang Y., Pan L., Wang Q., Han Y., Niu H., Shan D., Hoffmann A., Fang J. (2019). Induced Expression of Small Heat Shock Proteins Is Associated with Thermotolerance in Female *Laodelphax Striatellus* Planthoppers. Cell Stress Chaperones.

[38] Yang W.J., Xu K.K., Cao Y., Meng Y.L., Liu Y., Li C. (2019). Identification and Expression Analysis of Four Small Heat Shock Protein Genes in Cigarette Beetle, *Lasioderma Serricorne* (Fabricius). Insects.

[39] Kriehuber T., Rattei T., Weinmaier T., Bepperling A., Haslbeck M., Buchner J. (2010). Independent Evolution of the Core Domain and Its Flanking Sequences in Small Heat Shock Proteins. Faseb. J..

[40] Bakthisaran R., Tangirala R., Rao C.M. (2015). Small Heat Shock Proteins: Role in Cellular Functions and Pathology. Biochim. Biophys. Acta BBA Proteins Proteom..

[41] Perlman D., Halvorson H.O. (1983). A Putative Signal Peptidase Recognition Site and Sequence in Eukaryotic and Prokaryotic Signal Peptides. J. Mol. Biol..

[42] Lu M.X., Hua J., Cui Y.D., Du Y.Z. (2014). Five Small Heat Shock Protein Genes from *Chilo Suppressalis*: Characteristics of Gene, Genomic Organization, Structural Analysis, and Transcription Profiles. Cell Stress Chaperones.

[43] Shen Y., Gu J., Huang L.H., Zheng S.C., Liu L., Xu W.-H., Feng Q.-L., Kang L. (2011). Cloning and Expression Analysis of Six Small Heat Shock Protein Genes in the Common Cutworm, *Spodoptera Litura*. J. Insect Physiol..

[44] Huang L.H., Wang H.S., Kang L. (2008). Different Evolutionary Lineages of Large and Small Heat Shock Proteins in Eukaryotes. Cell Res..

[45] Savard J., Tautz D., Richards S., Weinstock G.M., Gibbs R.A., Werren J.H., Tettelin H., Lercher M.J. (2006). Phylogenomic Analysis Reveals Bees and Wasps (Hymenoptera) at the Base of the Radiation of Holometabolous Insects. Genome Res..

[46] Zahiri R., Kitching I.J., Lafontaine J.D., Mutanen M., Kaila L., Holloway J.D., Wahlberg N. (2011). A New Molecular Phylogeny Offers Hope for a Stable Family Level Classification of the Noctuoidea (Lepidoptera). Zool. Scr..

[47] Luo J., Cheng X.Y., Yan X., Tao W.Q., Holland J.D., Xu R.M. (2012). Characterization and Polymorphism Analysis of Phosphoglucose Isomerase Gene in the Fall Webwomt (*Hyphantria Cunea*). Bull. Entomol. Res..

[48] Jhan P.K., Lee K.Y. (2022). Developing Extreme Heat Acclimation in *Bemisia Tabaci* Mediterranean (Hemiptera: Aleyrodidae). Arch. Insect Biochem. Physiol..

[49] Queitsch C., Sangster T.A., Lindquist S. (2002). Hsp90 as a Capacitor of Phenotypic Variation. Nature.

[50] Concha C., Edman R.M., Belikoff E.J., Schiemann A.H., Carey B., Scott M.J. (2012). Organization and Expression of the Australian Sheep Blowfly (*Lucilia Cuprina*) Hsp23, Hsp24, Hsp70 and Hsp83 Genes. Insect Mol. Biol..

[51] Feliciello I., Akrap I., Ugarković Đ. (2015). Satellite DNA Modulates Gene Expression in the Beetle *Tribolium Castaneum* after Heat Stress. PLOS Genet..

[52] Liu W.W., Yang P., Chen X.M., Xu D.L., Hu Y.H. (2014). Cloning and Expression Analysis of Four Heat Shock Protein Genes in *Ericerus Pela* (Homoptera: Coccidae). J. Insect Sci..

[53] Regier J.C., Mazur G.D., Kafatos F.C. (1980). The Silkmoth Chorion: Morphological and Biochemical Characterization of Four Surface Regions. Dev. Biol..

[54] Yang C.L., Meng J.Y., Zhou L., Yao M.S., Zhang C.Y. (2021). Identification of Five Small Heat Shock Protein Genes in *Spodoptera Frugiperda* and Expression Analysis in Response to Different Environmental Stressors. Cell Stress Chaperones.

[55] Xie J., Xiong W., Hu X., Gu S., Zhang S., Gao S., Song X., Bi J., Li B. (2018). Characterization and Function Alanalysis of Hsp21.8b: An Orthologous Small Heat Shock Protein Gene in *Tribolium Castaneum*. J. Appl. Entomol..

[56] Chang Y.-W., Zhang X.-X., Lu M.-X., Du Y.-Z., Zhu-Salzman K. (2019). Molecular Cloning and Characterization of Small Heat Shock Protein Genes in the Invasive Leaf Miner Fly, *Liriomyza Trifolii*. Genes.

[57] Tao R., Li H., Sun Y.H., Yu X.H., Zhu H., Hao D.J. (2019). Indentification and Screening of Internal Reference Genes of *Hyphantria Cunea* (Lepidoptera: Arctiidae). Sci. Silvae Sin..

[58] Livak K.J., Schmittgen T.D. (2001). Analysis of Relative Gene Expression Data Using Real-Time Quantitative PCR and the 2(-Delta Delta C(T)) Method. Methods Companion Methods Enzymol..

