# Supplementary material for: Spatiotemporal Patterns of Five Small Heat Shock Protein Genes in Hyphantria cunea in Response to Thermal Stress
Source: Int J Mol Sci. 2023 Oct 14;24(20):15176. doi: 10.3390/ijms242015176 (PMC10606853; doi:10.3390/ijms242015176)
Supplement: Supplementary file 1 [file ijms-24-15176-s001.zip › ijms-2626526-supplementary.pdf]

Table S1. The sequence information used for sHSP multiple sequence alignments.

H.cunea Gene	Abbreviation	Gene Name	Species	Accession number	Identity
HcHSP18.9	BbHSP19.4	heat shock protein 19.4	Biston betularia	ADO33017.1	78.75%
	MsHSP19.3	heat shock protein 19.3	Mythimna separata	ATN45241.1	
	GmHSP18.9	small heat shock protein 18.9	Grapholita molesta	AKS40082.1	
	CsHSP19.0	heat shock protein 19.0	Chilo suppressalis	QYR68970.1	
HcHSP20.1	MsHSP19.5	heat shock protein 19.5	Mythimna separata	ATN45242.1	80.56%
	BmHSP19.5	19.5 kDa heat shock protein	Bombyx mori	NP_001164470.2	
	EhHSP19.7	heat shock protein 20-1	Eogystia hippophaecolus	AYA93247.1	
	ApHSP19.5	heat shock protein 19.5	Antheraea pernyi	APX61064.1	
HcHSP21.5	BmHSP20.8	heat shock protein hsp20.8	Bombyx mori	NP_001091794.1	83.79%
	AsHSP20.8	heat shock protein sHSP 20.8	Actias selene	ALI87024.1	
	GmHSP21.4	small heat shock protein 21.4	Grapholita molesta	AKS40075.1	
	GpHSP21.6	heat shock protein 21.6	Glyphodes pyloalis	QGZ00461.1	
HcHSP21.4	SiHSP21.4	heat shock protein 21.4	Sesamia inferens	AJA32863.1	99.47%
	HaHSP21.4	heat shock protein hsp21.4	Helicoverpa armigera	AGC39039.1	
	BmHSP21.4	heat shock protein hsp21.4	Bombyx mori	NP_001036985.1	
	GmHSP21.3	small heat shock protein 21.3	Grapholita molesta	AKS40074.1	
HcHSP29.8	SfHSP29.0	heat shock protein 29	Spodoptera frugiperda	QLR06860.1	71.19%
	OfHSP29.7	small heat shock protein Hsp29.7	Ostrinia furnacalis	UTU55755.1	
	BmHSP27.4	alpha-crystallin B chain-like	Bombyx mandarina	XP_028036235.1	
	NiHSP28.5	alpha-crystallin B chain-like	Nymphalis io	XP_050345805.1	

Table S2. The information on predicted tertiary structures of *H.cunea* sHSP genes.

Gene Name	Template	SMTL ID	Identity	GMQE	QMEAN	Substrate state	Specise
HcHSP18.9	ALPHA-CRYSTALLIN B	2wj7.1	48.86%	0.35	-0.61	Monomer	*Homo sapiens*
HcHSP20.1	HEAT SHOCK PROTEIN BETA-6	2wj5.1	39.44%	0.21	-1.17	Monomer	*Rattus norvegicus*
HcHSP21.5	ALPHA-CRYSTALLIN B	2y22.1	54.22%	0.29	-0.29	Monomer	*Homo sapiens*
HcHSP21.4	HEAT SHOCK PROTEIN BETA-6	2wj5.1	40.24%	0.26	-0.24	Monomer	*Rattus norvegicus*
HcHSP29.8	ALPHA-CRYSTALLIN B	2y22.1	32.43%	0.17	-0.6	Monomer	*Homo sapiens*

Table S3. The sequence information used for phylogenetic analysis of sHSPs.

Abbreviation	Gene Name	GeneBank No.	Species	Family
LdHSP21.6	heat shock protein beta-1	XP_023025087.1	*Leptinotarsa decemlineata*	Coleoptera
AgHSP21.6	heat shock protein beta-1 isoform X2	XP_012279730.1	*Anoplophora glabripennis*	Coleoptera
LserHSP21.8	heat shock protein 21.8	QWV59571.1	*Lasioderma serricorne*	Coleoptera
OaHSP21.5	heat shock protein beta-1 isoform X2	XP_012279730.1	*Orussus abietinus*	Hymenoptera
TcHSP21.5	heat shock protein beta-1 isoform X2	XP_024880994.1	*Temnothorax curvispinosus*	Hymenoptera
PpHSP21.7	small heat shock protein	EM45800.1	*Pteromalus puparum*	Hymenoptera
PxHSP21.6	small heat shock protein	AHW45924.1	*Plutella xylostella*	Lepidoptera
SfHSP21.4	heat shock protein	QNN88706.1	*Spodoptera frugiperda*	Lepidoptera
CsHSP21.4	heat shock protein 21.4	AGC23338.1	*Chilo suppressalis*	Lepidoptera
BdHSP20.6	heat shock protein hsp20.6	ARQ14799.1	*Bactrocera dorsalis*	Diptera
BdHSP23.8	heat shock protein hsp23.8	ARQ14802.1	*Bactrocera dorsalis*	Diptera
RpHSP22.1	heat shock protein 23-like	XP_036340981.1	*Rhagoletis pomonella*	Diptera
BdHSP23.0	heat shock protein hsp23.0	ARQ14801.1	*Bactrocera dorsalis*	Diptera
HcHSP16.0	small heat shock protein 16.0	QPZ75080.1	*Hyphantria cunea*	Lepidoptera
SfHSP29.0	heat shock protein	QNN88710.1	*Spodoptera frugiperda*	Lepidoptera
PxHSP19.2	small heat shock protein	AHW45925.1	*Plutella xylostella*	Lepidoptera
SfHSP19.1	heat shock protein	QQZ19789.1	*Spodoptera frugiperda*	Lepidoptera
SfHSP21.9	heat shock protein	QNN88707.1	*Spodoptera frugiperda*	Lepidoptera
HcHSP22.1	small heat shock protein 22.1	QPZ75082.1	*Hyphantria cunea*	Lepidoptera
CsHSP21.7	heat shock protein 21.7A	AGC23336.1	*Chilo suppressalis*	Lepidoptera
PxHSP21.8	small heat shock protein	AHW45926.1	*Plutella xylostella*	Lepidoptera
BdHSP21.6	heat shock protein hsp21.6	ARQ14800.1	*Bactrocera dorsalis*	Diptera
LsHSP21.3	heat shock protein Hsp21.3	ABE57138.1	*Liriomyza sativae*	Diptera
LsHSP21.7	heat shock protein Hsp21.7	ABE57139.1	*Liriomyza sativae*	Diptera
LsHSP19.5	heat shock protein Hsp19.5	ABE57140.1	*Liriomyza sativae*	Diptera
OcHSP21.1	heat shock protein 21.1	AJP36909.1	*Oxya chinensis*	Orthoptera
OcHSP20.7	heat shock protein 20.7	AJP36908.1	*Oxya chinensis*	Orthoptera
SgHSP20.7	heat shock protein 20.7	AEV89760.1	*Schistocerca gregaria*	Orthoptera
LmHSP20.7	heat shock protein 20.7	ABC84494.1	*Locusta migratoria*	Orthoptera
OcHSP20.4	heat shock protein 20.4	AJP36907.1	*Oxya chinensis*	Orthoptera
LmHSP20.5	heat shock protein 20.5	ABC84492.1	*Locusta migratoria*	Orthoptera
MhHSP22.0	putative small heat shock protein	ABM55532.1	*Maconellicoccus hirsutus*	Hemiptera
EpHSP21.7	heat shock protein 21.7	AGE92594.1	*Ericerus pela*	Hemiptera
LstrHSP22.0	small heat shock protein 22.0	AYP00114.1	*Laodelphax striatellus*	Hemiptera
LstrHSP21.4	small heat shock protein 21.4	AYP00112.1	*Laodelphax striatellus*	Hemiptera
LstrHSP21.2	small heat shock protein 21.2	AYP00111.1	*Laodelphax striatellus*	Hemiptera
LstrHSP20.1	small heat shock protein 20.1	AYP00110.1	*Laodelphax striatellus*	Hemiptera
LserHSP22.2	small heat shock protein 22.2	QDZ38466.1	*Lasioderma serricorne*	Coleoptera
LserHSP20.9	heat shock protein 21	QWV59569.1	*Lasioderma serricorne*	Coleoptera

LserHSP20.2	small heat shock protein 20.2	QDZ38462.1	*Lasioderma serricorne*	Coleoptera
AhHSP20.8	sHSP20.8	QNJ44844.1	*Agasicles hygrophila*	Coleoptera
AhHSP21.5	sHSP21.5	QNJ44846.1	*Agasicles hygrophila*	Coleoptera
AhHSP21.0	sHSP21	QNJ44844.1	*Agasicles hygrophila*	Coleoptera
PxHSP20.1b	small heat shock protein	AHW45918.1	*Plutella xylostella*	Lepidoptera
CsHSP24.3	heat shock protein 24.3	ASK86231.1	*Chilo suppressalis*	Lepidoptera
PxHSP20.1a	small heat shock protein	AHW45919.1	*Plutella xylostella*	Lepidoptera
SfHSP19.7	heat shock protein	QQZ19788.1	*Spodoptera frugiperda*	Lepidoptera
HcHSP20.0	small heat shock protein 20.0	QPZ75079.1	*Hyphantria cunea*	Lepidoptera
PxHSP23.4	small heat shock protein	AHW45920.1	*Plutella xylostella*	Lepidoptera
SfHSP20.1	heat shock protein	QNN88703.1	*Spodoptera frugiperda*	Lepidoptera
HcHSP20.3	small heat shock protein 20.3	QPZ75081.1	*Hyphantria cunea*	Lepidoptera
CsHSP19.0	heat shock protein 19.0	QYR68970.1	*Chilo suppressalis*	Lepidoptera
SfHSP19.4	heat shock protein	QNN88700.1	*Spodoptera frugiperda*	Lepidoptera
AcHSP22.6	sHSP22.6	AGX26086.1	*Apis cerana cerana*	Hymenoptera
McHSP23.8	small heat shock protein	ACF21815.1	*Macrocentrus cingulum*	Hymenoptera
AcHSP24.9	small heat shock protein	AEH05929.1	*Apis cerana cerana*	Hymenoptera

Table S4. The motif analysis of sHSP sequences.

Motif	Sites	Width	E-value	Logo
1	61	21	2.5e-618	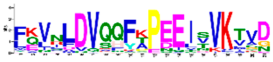
2	61	31	1.0e-996	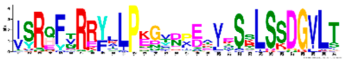
3	61	15	1.2e-517	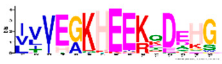
4	39	24	1.2e-376	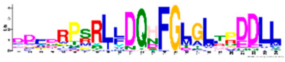
5	10	50	1.0e-349	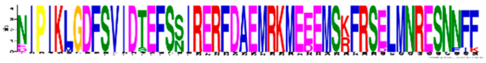
6	44	21	2.40E-280	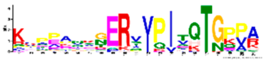
7	40	15	2.30E-195	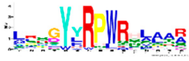
8	40	11	3.70E-97	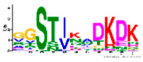
9	41	8	5.00E-84	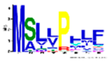
10	10	20	6.30E-81	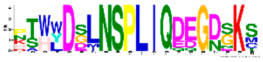
11	10	15	3.20E-49	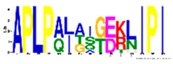
12	10	11	1.90E-27	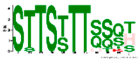

Table S5. Significance analysis of gene expression levels of *HcHSPs* at different developmental stages.

Developmental stage	HcHSP18.9	HcHSP20.1	HcHSP21.5	HcHSP21.4	HcHSP29.8
E	1.23±0.35d	36.37±9.26c	251.58±88.29bc	0.95±0.03d	4.93±0.59b
2L	1.08±0.32d	1.03±0.17e	1.04±0.22e	1.01±0.12d	1.02±0.14c
4L	0.58±0.04d	7.78±1.12d	5.2±1.04d	0.88±0.07d	0.23±0.03d
6L	0.64±0.04d	1.89±0.52e	1.82±0.16de	0.69±0.18d	0.04±0e
FP	48.01±3.66a	67.79±6.8bc	370.18±17.25b	3.81±0.67c	14.28±1.62a
MP	15.48±2.37b	401.72±40.92a	324.67±39.33b	5.27±0.5bc	13.53±0.49a
FA	5.36±0.86c	644.92±74.15a	8195.94±795.49a	8.94±1.13b	11.84±0.9a
MA	32.01±3.52ab	98.32±12.21b	77.14±12.27c	44.02±5.02a	23.58±4.97a

Table S6. Significance analysis of gene expression levels of *HcHSPs* at different developmental stages under heat stress conditions.

Developmental stage	Temperature	HcHSP18.9	HcHSP20.1	HcHSP21.5	HcHSP21.4	HcHSP29.8
E	25°C	1.1±0.31Ab	1.09±0.27Ab	1.21±0.42Ab	1±0.03Aa	1.01±0.12Ab
	30°C	3.82±1.26Aab	1.62±0.22BCb	0.23±0.08Dc	1.63±0.66Aa	1.29±0.24Cb
	35°C	6.52±0.32Bab	4.79±1.42Eb	0.99±0Cb	0.66±0.17Ba	0.62±0.14Cb
	40°C	12.54±3.42CDa	361.63±65.33Ba	10.29±0.58Da	2.68±1.21Aa	9.35±2.57ABa
	43°C	9.41±6.03Bab	193.22±24.76Ea	5.71±1.57Da	1.77±0.72Aa	0.71±0.49Db
2L	25°C	1.08±0.32Ab	1.03±0.17Ad	1.07±0.29Ad	1.01±0.12Aa	1.02±0.14Ac
	30°C	1.8±0.08Ab	5.33±0.17Ac	4.47±0.29Ac	1.07±0.09Aa	2.69±0.72ABCbc
	35°C	8.69±0.68Ba	199.33±14.59Ab	67.36±0.32Ab	1.21±0.08Aa	6.08±0.11Aab
	40°C	6.41±2.1Da	2459.41±1103.95Aa	995.15±474.68Aa	0.23±0.02Cb	9.08±3.7ABa
	43°C	7.53±1.09Ba	658.67±23.26Da	484.81±46.69Ba	0.2±0.03Bb	1.35±0.14CDc
4L	25°C	1±0.06Ae	1.02±0.14Ad	1.04±0.2Ad	1.01±0.1Ab	1.02±0.17Ad
	30°C	2.47±0.18Ad	1.29±0.23BCd	1.22±0.1BCd	1.09±0.04Aab	4.1±0.88Ac
	35°C	7.61±0.74Bc	117.13±20.63ABc	14.99±2.7Bc	1.41±0.05Aa	7.88±0.44Ab
	40°C	37.22±1.56Bb	526.15±29.27Bb	153.74±13.7Bb	1.13±0ABab	13.6±0.97Aab
	43°C	109.37±13.98Aa	1347.29±127.04BCa	472.29±43.27Ba	1.17±0.1Aab	21.02±2.32Aa
6L	25°C	1±0.07Ac	1.07±0.29Ad	1±0.09Ae	1.02±0.16Aa	1.02±0.15Ac
	30°C	3.24±1.09Abc	2.29±0.43Bd	3.04±0.99ABd	1.13±0.15Aa	3.18±0.22ABb
	35°C	6.5±1.01Bb	84.55±12.25BCc	46.43±5.59Ac	1.09±0.02Aa	4.25±0.75ABb
	40°C	10.96±3.54CDb	478.64±10.05Bb	602.75±58.89Ab	0.92±0.07Ba	17.52±4.43Aa
	43°C	67.06±26.07Aa	3343.04±842.47Aa	1935.61±489.89Aa	1.93±0.56Aa	39.01±10.7Aa
FP	25°C	1±0.07Ac	1.01±0.1Ad	1±0.04Ad	1.03±0.18Aa	1.01±0.11Ad
	30°C	2.41±0.3Ab	1.35±0.14BCd	5.34±0.23Ac	1.01±0.04Aa	2.37±0.17ABCc
	35°C	7.28±0.68Ba	27.43±0.58Dc	17.75±1.74Bb	0.89±0.03ABa	5.9±0.95Ab
	40°C	9.1±0.32Da	350.54±12.6Bb	63.54±1.69BCa	0.88±0.05Ba	9.35±0.18Aa
	43°C	8.19±0.47Ba	671.22±17.57CDa	59.2±4.92Ca	0.54±0.03ABb	3.63±0.21BCc
MP	25°C	1.02±0.15Ad	1.01±0.1Ad	1.01±0.12Ad	1±0.09Aa	1±0.03Ad
	30°C	2.2±0.31Ad	1.4±0.12BCd	3.28±0.68ABc	0.99±0.1Aa	2.83±0.31ABCc
	35°C	9.33±1.09Bc	43.01±3.06CDc	15.43±0.74Bb	1.04±0.01ABa	6.83±0.34Ab

	40°C	30.58±7.15BCb	763.68±102.16ABb	54±1.77Ca	0.94±0.02Ba	7.71±0.85ABb
	43°C	76.97±5.08Aa	1652.55±60.2ABa	80.78±5.97Ca	1±0.11Aa	12.03±1.06ABa
FA	25°C	1.02±0.16Ad	1.01±0.11Ac	1±0.09Ab	1.01±0.12Aa	1±0.07Ac
	30°C	2.84±0.06Ac	1.05±0.18Cc	1.02±0.17Cb	0.95±0.04Aa	1.77±0.1ABCb
	35°C	20.76±3.14Ab	4.23±0.17Eb	1.4±0.1Cb	1.28±0.17Aa	2.19±0.2Bab
	40°C	112.66±5.96Aa	92.25±17.53Ca	4.04±0.19Ea	0.98±0.01Ba	2.63±0.3Cab
	43°C	111.13±26.27Aa	145.71±25.33Ea	4.54±0.97Da	0.81±0.12Aa	2.81±0.15Ca
MA	25°C	1.01±0.11Ad	1.01±0.12Ac	1.02±0.16Ad	1.01±0.11Aa	1.04±0.21Ab
	30°C	1.42±0.26Ad	1.29±0.2BCc	5.04±0.38Ac	1.57±0.23Aa	1.5±0.39BCab
	35°C	23.78±4.12Ac	51.37±14.18CDb	71.9±16.66Ab	1.27±0.13Aa	2.48±0.53Bab
	40°C	136.45±6.75Aa	1980.18±76.99Aa	648.79±12.53Aa	1.56±0.11ABa	3.22±0.39BCa
	43°C	68.41±6.42Ab	1855.56±252.54ABa	527.45±58.31Ba	1.27±0.13Aa	1.82±0.33Cab

Note: Different lowercase letters indicate significant differences among the different temperature treatments at the same developmental stage, and capital letters are used for different developmental stages at the same temperature. Significance was determined via analysis of variance ($p < 0.05$).

Table S7. Significance analysis of gene expression levels of *HcHSPs* in different tissues before and after being subject to heat stress conditions.

Treatment	Tissue	HcHSP18.9	HcHSP20.1	HcHSP21.5	HcHSP21.4	HcHSP29.8
25°C	Gut	0.14±0.02bc	0.38±0.17ab	5.15±1.79ab	4.67±1.12ab	0.04±0.01bc
	Malpighian tube	0.06±0.03bcd	0.13±0.04bc	0.59±0.23cd	7.36±1.61ab	0.57±0.32a
	Silk gland	0.01±0d	0.05±0.01c	0.17±0.08d	4.68±2.09ab	0±0c
	Hemolymph	0.03±0.01cd	0.14±0.04bc	21.36±7.1a	2.1±0.47b	0.02±0bc
	Fat body	0.29±0.09ab	0.23±0.04abc	1.51±0.34bc	5.54±0.98ab	0.04±0.01ab
	Cuticle	1.11±0.18a	0.76±0.13a	2.37±0.48bc	13.86±4.11a	0.5±0.14a
43°C	Gut	13.69±1.92C ***	218.40±87.08B ***	566.36±137.63 AB ***	5.33±1.06BC ns	0.29±0.06C *
	Malpighian tube	59.61±20.25B ***	270.8±34.07B ***	762.97±42.22 AB ***	8.23±0.42B ns	3.81±0.11AB *
	Silk gland	20.84±2.84BC ***	182.81±19.64B ***	327.8±12.12 bc ***	5.77±0.68BC ns	0.45±0.07CD ***
	Hemolymph	32.37±6.09BC ***	170.56±48.72B ***	596.51±221.03 AB **	3.04±0.4C ns	1.77±0.43BC ***
	Fat body	25.49±3.75BC ***	241.75±7.664B ***	502.97±19.51B ***	11.37±1.44B *	2.97±0.576B ***
	Cuticle	638.72±228.09A ***	971.42±229.88A ***	1381.07±276.81 A ***	48.22±11.21A *	14.53±6.92A **

Note: Different lowercase letters indicate significant differences between different tissues under 25°C treatment, and capital letters are used for significant differences between different tissues under 43°C treatment ($P < 0.05$). The significant differences between 25°C control treatments and 43°C treatments in the same tissue are indicated by an asterisk (*), corresponding to $P < 0.05$ level (*), $P < 0.01$ level (**), and $P < 0.001$ level (***), while ns indicates results that are not significant.
